# Visualization and Quantitative Analysis of Reconstituted Tight Junctions Using Localization Microscopy

**DOI:** 10.1371/journal.pone.0031128

**Published:** 2012-02-02

**Authors:** Rainer Kaufmann, Jörg Piontek, Frederik Grüll, Manfred Kirchgessner, Jan Rossa, Hartwig Wolburg, Ingolf E. Blasig, Christoph Cremer

**Affiliations:** 1 Kirchhoff Institute for Physics, University of Heidelberg, Heidelberg, Germany; 2 Leibniz-Institut für Molekulare Pharmakologie (FMP), Berlin, Germany; 3 Institute of Pathology and Neuropathology, University Medical School Tübingen, Tübingen, Germany; 4 Institute of Molecular Biology, Mainz, Germany; 5 Institute for Pharmacy and Molecular Biotechnology, University of Heidelberg, Heidelberg, Germany; University Hospital Hamburg-Eppendorf, Germany

## Abstract

Tight Junctions (TJ) regulate paracellular permeability of tissue barriers. Claudins (Cld) form the backbone of TJ-strands. Pore-forming claudins determine the permeability for ions, whereas that for solutes and macromolecules is assumed to be crucially restricted by the strand morphology (i.e., density, branching and continuity). To investigate determinants of the morphology of TJ-strands we established a novel approach using localization microscopy.

TJ-strands were reconstituted by stable transfection of HEK293 cells with the barrier-forming Cld3 or Cld5. Strands were investigated at cell-cell contacts by Spectral Position Determination Microscopy (SPDM), a method of localization microscopy using standard fluorophores. Extended TJ-networks of Cld3-YFP and Cld5-YFP were observed. For each network, 200,000 to 1,100,000 individual molecules were detected with a mean localization accuracy of ∼20 nm, yielding a mean structural resolution of ∼50 nm. Compared to conventional fluorescence microscopy, this strongly improved the visualization of strand networks and enabled quantitative morphometric analysis. Two populations of elliptic meshes (mean diameter <100 nm and 300–600 nm, respectively) were revealed. For Cld5 the two populations were more separated than for Cld3. Discrimination of non-polymeric molecules and molecules within polymeric strands was achieved. For both subtypes of claudins the mean density of detected molecules was similar and estimated to be ∼24 times higher within the strands than outside the strands.

The morphometry and single molecule information provided advances the mechanistic analysis of paracellular barriers. Applying this novel method to different TJ-proteins is expected to significantly improve the understanding of TJ on the molecular level.

## Introduction

Tight junctions (TJ) form the paracellular barrier in epithelia and endothelia. They limit and regulate the paracellular permeation of ions, solutes and macromolecules [1]. TJ appear as an anastomosing network of strands composed of transmembrane proteins (freeze-fracture electron microscopy) [2] and as fusions of the membranes of two neighboring cells (transmission electron microscopy) [3]. The tetraspan membrane proteins of the claudin (Cld) family constitute the polymeric backbone of TJ by *cis*-interactions (side-by-side, along the membrane) and *trans*-interactions (head-to-head, between opposing cells) [4], [5]. The claudin subtypes are expressed in a tissue-specific manner [6] and can be functionally divided in barrier-forming claudins (e.g. Cld1, −3, −5), and pore-forming claudins (e.g., Cld2, −10, −15).

The control of paracellular ion permeability by pore-forming claudins is well characterized [1]. In contrast, the regulation of paracellular permeability for solutes and macromolecules is less understood. The density and branching of TJ-strands and the dynamics of their assembly and disassembly are assumed to determine the extent of paracellular permeation [7], [8]. The dynamics of TJ-proteins can be analyzed by fluorescence recovery after photobleaching (FRAP) [9], [10], [11]. The morphology of the TJ-network is usually analyzed by freeze-fracture electron microscopy (FF-EM). This powerful technique provides very high spatial resolution (in the nm order) and TJ-strands can be identified due to their unique strand-like arrangement of intramembranous particles. Despite its high structural resolution, FF-EM has the following drawbacks: (i) TJ-proteins can be visualized by morphological features, but their molecular identification has to be performed by additional immunolabeling with much lower resolution; (ii) Due to separation of protoplasmatic- (P) and exoplasmatic- (E) face of the membrane, not all molecules in the strand are detected in one replica; (iii) Non-polymerized TJ-proteins and other intramembranous particles cannot be distinguished; and (iv) Quantitative morphometric analysis is very difficult. Therefore, alternative methods to detect TJ-strands and to investigate their morphology and composition are desirable.

Previously, we optimized a cellular system to reconstitute TJ-strands by transfection of HEK293 cells with claudin constructs. In this system, the TJ-strands consist of a defined claudin backbone but not of additional tight junction associated proteins, e.g., zonula occludens proteins. This provides conditions in which self-assembly characteristics of claudins can be analyzed in a native membrane environment. Up to now, the HEK293 cell system was used to identify determinants of claudin-claudin interaction [5], interaction of claudins with extracellular ligands [12] and heterophilic compatibility between claudin subtypes [11]. The morphology of the resulting strands was analyzed by FF-EM [5], [11]. However, a limiting factor was the quantitative analysis of a sufficient number of TJ-strands.

To improve the visualization of networks of reconstituted TJ-strands (TJ-networks) and the morphometric analysis on the nanoscale, we applied localization microscopy. This technique is based on the individual detection of optically isolated molecules. Imaging extended biological structures with a structural resolution in the 50 nm range was first performed by using photoactivatable or photoswitchable fluorophores [13], [14], [15]. Recently developed methods enabled localization microscopy using standard fluorophores [16], [17], [18]. In the present report, we used Spectral Position Determination Microscopy (SPDM) in accordance with Lemmer et al. [17]. This technique is very suitable for the analysis of TJ-strands, given that it enables the use of claudin-YFP-transfected cells that were extensively characterized by other methods [11].

Compared to other cellular polymers like microtubules, TJ-strands cannot be reconstituted *in vitro*. Therefore, the applicability of the localization microscopy method to detect TJ-strands in the apical plasma membrane is important and must not be restricted to the basal membrane like TIRF-based methods. Localization microscopy not only offers an enhanced structural resolution (∼50 nm in the present measurements) compared to conventional fluorescence microscopy (>200 nm), it also provides single molecule information which can be used for statistical analyses.

In this report, reconstituted TJ-strands in cells were visualized with super-resolution by localization microscopy (SPDM). A quantitative analysis was performed to investigate their morphology which strongly contributes to the barrier properties of TJ.

## Results

### Visualisation of tight junction strands with standard and super-resolution fluorescence microscopy

**Figure 1:**
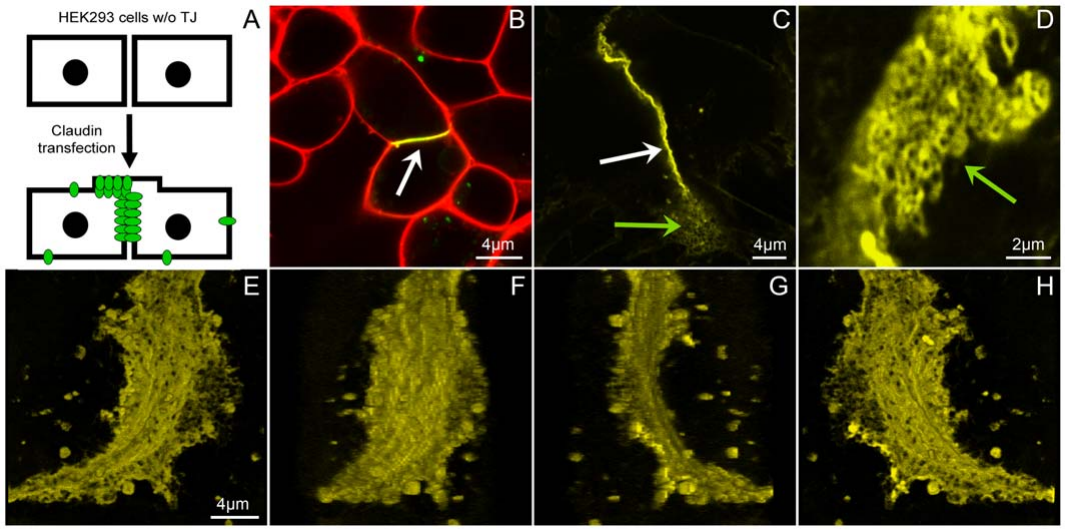
Reconstitution of TJ-strands formed by claudin proteins. **A**: Schema of reconstitution of TJ-strands. HEK293 cells without endogenous TJ are transfected with claudins. TJ-strands are formed at contacts between claudin-expressing cells in the lateral membrane and at apical cellular protrusions. Confocal images of living (**B**) or fixed (**C**–**H**) cells. **B**: Cld3-YFP (green) is enriched at contacts between two claudin-expressing cells (white arrow). Plasma membrane (red) of living cells was labeled with CellMask™ Deep Red (Invitrogen, 1.25 µg/ml CellMask™ Deep Red in Hank's Buffered Salt Solution for 20 min). **C**: Cld3-YFP strands in the lateral membrane running in z-direction along the optical axis (white arrow). Cld3-YFP strands running in the apical membrane in xy-direction in the object plane (green arrow). **D**: Higher magnification of Cld3-YFP strands running in xy-direction (green arrow). **E**–**H**: Sequential images of a 3D projection of Cld3-YFP at cell-cell contact (angle: 0°, 50°, 140°, 200°).

TJ-strands were reconstituted by transfection of HEK293 cells with Cld3-YFP (Fig. 1A) and are formed at cell-cell contacts between HEK293 cells expressing Cld3-YFP [11], [12]. Figure 1 shows an example for the reconstitution of TJ networks formed by claudin proteins detected by conventional confocal microscopy. Strands oriented in the z-direction in the lateral membrane at cell-cell contacts appear as thin structures with high intensity values of the YFP-signals in the projected images in xy-direction (Fig. 1B, 1C) [5]. In addition, cells are growing partly on top of each other at cell-cell contacts (Fig. 1A). There, strands oriented in xy-direction in the plasma membranes are detected from the top (Fig. 1D).

**Figure 2:**
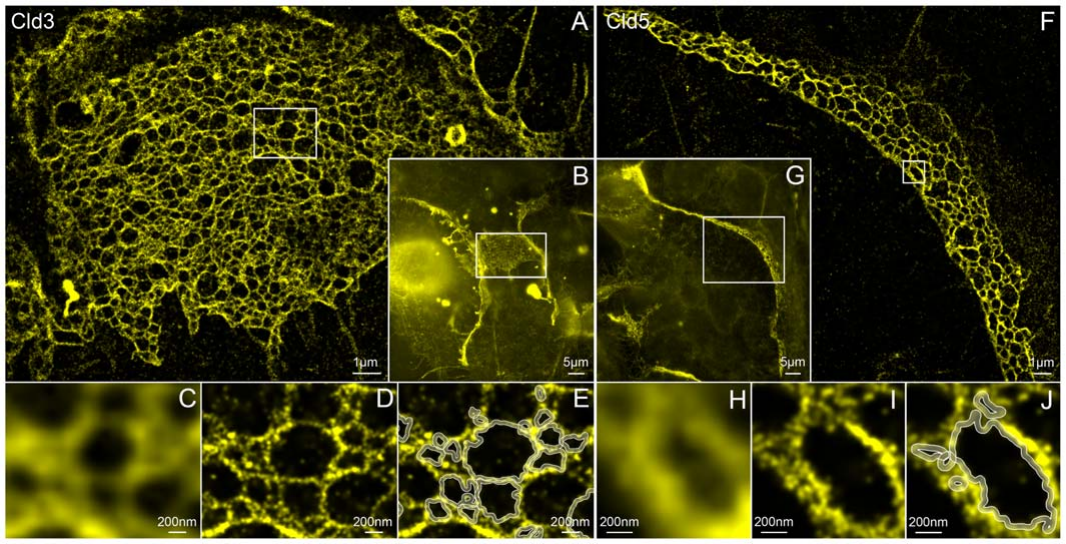
Tight junction networks formed by Cld3-YFP and Cld5-YFP. **A**: Localization microscopy image of the region marked in the conventional wide-field fluorescence image (**B**) of YFP labeled Cld3 in HEK293 cells. More than 450,000 molecules were detected with a mean localization accuracy of ∼20 nm. The mean distance to the next neighboring molecule in the image is ∼10 nm, thus the mean effective optical resolution is only limited by the localization accuracy and yielding ∼48 nm. Magnified images of the region marked in **A** are shown in **D** and **E**. Mesh-like structures identified and analyzed by the algorithm are indicated in white. **C** represents the same region but taken from the conventional wide-field fluorescence image. **F**–**J**: Analogue visualization of Cld5-YFP expressed in HEK293 cells. Here, ∼260,000 molecules were detected with a mean localization accuracy of ∼21 nm. The mean distance to the next neighboring molecule in this image is ∼7 nm; yielding a structural resolution of ∼50 nm.

For super-resolution imaging, HEK293 cells stably expressing Cld3-YFP were fixed, and cell-cell contacts with enrichment of Cld3-YFP indicating strands were identified by conventional wide-field fluorescence microscopy (Fig. 2B). The network for TJ-strands can only be resolved roughly by conventional fluorescence microscopy. Only large loops with a diameter above the conventional optical resolution can be identified (Fig. 2C). Using localization microscopy, the network of strands can be imaged in much more detail (Fig. 2A, 2D). The strands were spanning areas in the range of 200 to 400 µm^2^. Only signals in focus (∼600 nm in z-direction) were efficiently detected. Therefore, strands along the lateral plasma membrane were not visualized. In the localization microscopy measurements of Cld3, a total amount of single molecules in the range of 450,000 to 1,100,000 was detected for each TJ-network. The mean localization accuracy was ∼21 nm, and the mean distance to the next neighboring molecule in the images was ∼10 nm. Thus, the mean effective optical resolution (structural resolution) was only limited by the localization accuracy and yielded ∼50 nm. The magnified images (Fig. 2C–E, 2H–J) clearly demonstrate the difference in structural resolution. Strands and the meshes formed by the strands can be identified (Fig. 2E, 2J) and analyzed by an algorithm introduced below.

To compare TJ-strands formed by Cld3 with those formed by Cld5, HEK293 cells stably expressing Cld5-YFP were fixed and also analyzed by SPDM. Similar to Cld3-YFP, extended networks of TJ-strands spanning areas in the range of ∼100 µm^2^ were imaged (Fig. 2F–J). In each TJ-network, 220,000 to 290,000 molecules were detected with a mean localization accuracy of ∼21 nm. As for the measurements of Cld3-YFP, the structural resolution was also only limited by the localization accuracy resulting in ∼50 nm.

**Figure 3:**
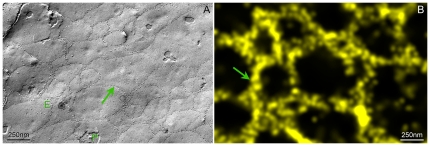
Networks of TJ-strands and meshes visualized by freeze fracture EM and localization microscopy. **A**: Freeze-fracture EM of Cld5-YFP/Cld3-cotransfected HEK293 cells. **B**: Localization microscopy of Cld5-YFP transfected HEK293 cells. In freeze fracture EM the Cld5/Cld3-strands appear as chains of 10 nm intramembranous particles (arrow) on the exoplasmic face (E) and protoplasmic face face (P) of the plasma membrane. Due to lower resolution of localization microscopy the strands appear thicker (arrow).

The networks of TJ-strands detected by localization microscopy were similar to those detected by FF-EM of HEK293 cells transfected with Cld5-YFP [5], Cld3 [11] or Cld5-YFP/Cld3 (Fig. 3).

### Algorithm for the quantitative analysis of mesh-like structures

The analysis of the mesh-like structures has been performed on localization microscopy images with a pixel size of 2 nm. Since the network of TJ-strands can be resolved by SPDM with a structural resolution of ∼50 nm, an algorithm to quantify morphological parameters of the network was developed.

**Figure 4:**
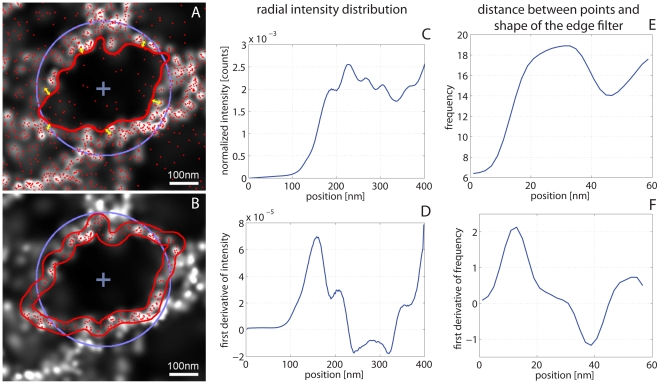
Analysis of mesh-like structures. **A**,**B**: Local point densities are visualized as grey values in the localization microscopy image. Positions of the detected single molecules are indicated by red dots. **C**: Radial intensity distribution of the mesh-like structure shown in **A**,**B**. **D**: First derivative of the radial intensity distribution. The first zero-crossing marks the position of the first maximum of the intensity distribution. A blue circle in **A**,**B** represents the approximation by the circle with a radius corresponding to the maximum of the radial intensity distribution. **E**: Histogram of the distances between the single molecule positions and the shape of the edge filter (indicated by yellow arrows in **A** for some of the positions). **F**: The first zero-crossing of the first derivative gives the distance of the majority of the points. This value is used to correct the underestimated size of the mesh obtained by the edge filter (red line in **A**). In **B** the corrected shape is illustrated by the two red lines. The area in between is used to determine the molecule density on the strand of the mesh.

As a first step of the analysis of the mesh-like structures their rough extension is estimated by looking at the radial intensity distribution starting from their manually determined centre. The mean radius 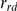 of a mesh is given by the position of the distribution's maximum (Fig. 4C, 4D). For the second step a region of interest (ROI) with the size of 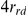 is set around the mesh. A Canny edge filter (thresholds: low: 0.04, high: 0.1; STD of Gaussian filter: 10 nm) is used to get a more precise representation of the real shape that might be more complex. The actual size of a mesh is systematically underestimated by the edge filter (Fig. 4A). Therefore, all the distances between the molecule positions within the ROI and the shape of the edge filter are determined. The maximum in the histogram of the distances indicates the difference between the result of the edge filter and the real shape of the mesh (Fig. 4E, 4F). After correction (Fig. 4B), the circumference 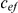 can be determined very precisely. The mean diameter of a mesh is calculated by using the circumference and the approximation of circular shape (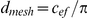) for a better comparison with the structures visible in the images. The convolution of the shape with a circular area with a diameter corresponding to the width of the strands of the mesh-like structures yields the area of the mesh. The width of the claudin strands is assumed to be ∼10 nm. For a high point density in the image the width of the strands is given by the localization accuracy 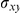, which is used for the radius of the circular area. Now, the density of molecules on the strands of a mesh can be determined. Also other information like the Feret diameter and the density of the molecules inside a mesh-like structure can be gathered.

### Cld3 and Cld5 networks consist of two populations of meshes with different diameters

**Figure 5:**
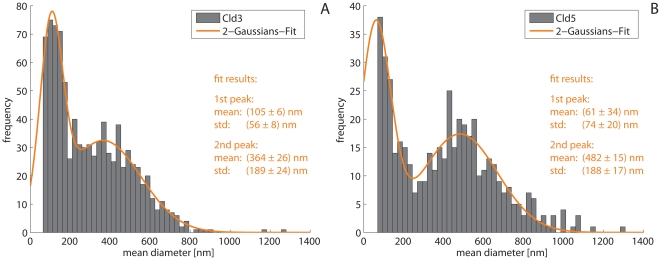
Diameter of the mesh-like structures. **A**: Size measurements of Cld3 tight junction networks. The histogram of diameters shows one distribution for very small sizes (diameter of ∼100 nm) overlaying a second distribution for a diameter of ∼360 nm. Results of the measurements of Cld5 are depicted in **B**. The histogram also shows two distributions (∼60 nm and ∼480 nm). Both data sets were fitted with the sum of two Gaussians. The results of the fits are plotted in orange; fitted parameters are given including standard errors.

The histograms of the diameters of the meshes show two different distributions for Cld3 as well as for Cld5 (Fig. 5). In both cases a distribution of diameters in the range of ∼400 nm is clearly visible. For Cld3 a mean value of 364 nm and a FWHM (full width at half maximum) of 444 nm were obtained; for Cld5 a mean value of 482 nm with a FWHM of 442 nm. Besides these large meshes, a distribution of very small ones with diameters around 100 nm and below was observed. For the Cld3, a Gaussian fit yielded a mean diameter of 105 nm with a FWHM of 132 nm. The results of the fit for the Cld5 measurements yielded a mean diameter of 61 nm with a FWHM of 174 nm. For the distribution of small meshes formed by Cld5, the inaccuracy of the fit result was rather high because the mean value of the distribution appears to be below the size of structures that could be resolved and analyzed. Therefore, only a part of the distribution is described by the results of the measurements in the histogram which leads to only a vague fit.

In sum, partially different results were obtained for both claudins. The two distributions of diameters for the mesh-like structures formed by Cld5 differ more from each other compared to those of Cld3. This significant difference (Kolmogorov-Smirnov test, p<0.001) suggests that in the case of Cld5 there are more very small (<100 nm) and more large meshes (∼500 nm) as in the TJ-networks of Cld3. For the latter, the two distributions of diameters are more overlapping.

### Cld3 and Cld5 form slightly elongated meshes oriented preferentially parallel to the network

**Figure 6:**
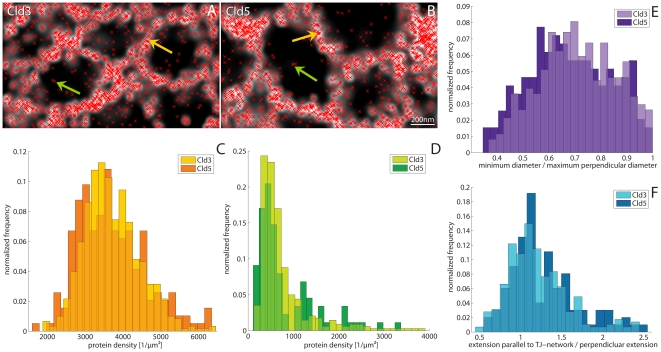
Densities of detected proteins and shape of the mesh-like structures. **A**,**B**: Localization microscopy images of Cld3-YFP (**A**) and Cld5-YFP (**B**) with overlay of single molecule positions represented by red crosses. **C**: Histogram of the density of detected proteins on the strands of the mesh-like structures (indicated in **A**,**B** by orange arrows). Both protein types show similar distributions with mean values of ∼3700 proteins/µm^2^. The standard deviation of the distribution for Cld3 is 760 proteins/µm^2^. The distribution for Cld5 is wider and provides a standard deviation of 900 proteins/µm^2^. **D**: Histogram of the density of detected proteins beside the strands of the meshes (indicated in **A**,**B** by green arrows). Here, the distribution for Cld3 and Cld5 are very similar, too (mean values: ∼780 proteins/µm^2^ with STDs of ∼640 proteins/µm^2^). For many meshes (especially the very small ones) no proteins could be detected on their inside. These are not considered in the histograms. **E**: Histograms of the minimum diameter divided by its maximum perpendicular diameter of the meshes. Both protein types show similar distributions with a maximum at ratio of ∼0.7. **F**: Histograms of the extension of the meshes parallel to the orientation of the whole TJ-network (principal axis) divided by its perpendicular extension showing that the mean orientation of the meshes of both protein types is parallel to the orientation of the whole TJ-network (mean value for Cld3: ∼1.21 with STD: ∼0.38; mean value for Cld5: ∼1.29 with STD: ∼0.42). All histograms are normalized to the total amount of analyzed meshes.

The analysis of the Feret diameter (minimum diameter / maximum perpendicular diameter) yielded very similar results for Cld3 and Cld5 meshes. The histograms in Figure 6E show maxima at a ratio of ∼0.7 which indicates that most of the meshes are not circular but are elongated in one direction. This feature seems to be slightly more pronounced for Cld5 compared to Cld3. An analysis of the extensions of the meshes with respect to the direction of the whole TJ-network revealed that, on average, the elliptically shaped meshes are oriented parallel to the extension (longer axis) of the network (Fig. 6F, Fig. S3). To get a more accurate result only meshes with a diameter >200 nm were investigated, because their morphology can be determined very precisely. The mean ratio between the extension of the meshes in the direction of the network and the perpendicular extension yielded a value of 1.21 (STD: 0.38) for Cld3 and 1.29 (STD: 0.42) for Cld5.

### Protein density in the TJ-networks is similar for Cld3 and Cld5

The absolute number and density of molecules cannot be determined since not every fluorophore is detected during the acquisition time. Nevertheless, relative comparisons of the number of detected molecules and densities between samples and between different areas of one sample are possible.

Figure 6C shows histograms of the densities of detected proteins on the strands of the mesh-like structures which were previously analyzed with respect of their diameters. Both Cld3 and Cld5 revealed a mean density of detected proteins of ∼3700 proteins/µm^2^. Although the distribution for Cld5 was slightly wider, both protein types show very similar results. In addition to the analysis of the protein density on the strands of a mesh, the density of detected proteins inside the meshes (off the strands) was determined (Fig. 6D). The analysis revealed a mean density of ∼780 proteins/µm^2^ for both of the claudin subtypes. This indicates that the molecule density within the strands is ∼5 times higher than outside the strands. However, due to the structural resolution, it must be considered that the apparent width of the strands is in the range of 50 nm (due to the scattering of the molecule positions around the actual structure because of the limited localization accuracy). In contrast, EM indicates a width of ∼10 nm [5]. Taking these results into account, the molecule density within the polymeric strands is estimated to be ∼24 times higher than that of the non-polymerized molecules, where the structural resolution does not influence the density of detected molecules.

**Figure 7:**
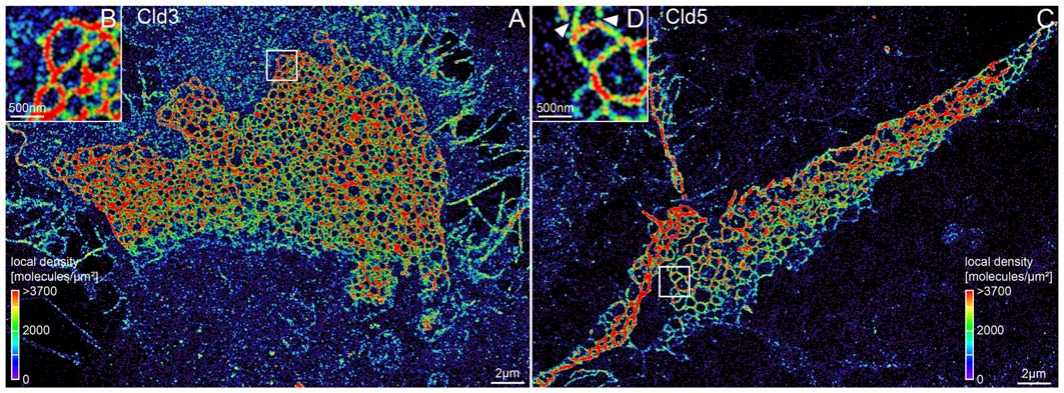
Visualization of local densities of detected proteins in and around TJ-networks formed by Cld3 and Cld5. **A**: Localization microscopy image of Cld3-YFP labeled HEK293 cells. The local density was determined within a radius of 50 nm for every detected molecule and color coded in the image. The dynamic range was chosen between 0 and 3700 molecules/µm^2^ - the mean density of detected molecules on the strands of the analyzed meshes. **B**: Magnified image of the region marked in **A**. **C**,**D**: Analogue visualization of Cld5-YFP. Gaps in the meshes are indicated by white arrowheads in **D**.

In Figure 7, the local protein densities on the TJ-networks of Cld3 and Cld5 are visualized in the localization microscopy images by color coding. The local densities were determined within a radius of 50 nm around each detected molecule. The dynamic range was chosen between 0 and 3700 molecules/µm^2^ - the mean density of detected molecules on the strands of the analyzed meshes. This enables a visual comparison of different local protein densities beside the strands formed by Cld3 and Cld5. Areas with distinct local protein densities were detected for Cld3 and for Cld5. For example, in the image shown in Figure 7A the area above the TJ-network shows higher local densities than the area below the TJ-network. Furthermore, for the Cld5 image shown in Figure 7C the areas around the TJ-network show lower local molecule densities than those obtained for Cld3. For both claudins, additional focal enrichments in the local density outside of the networks were found. The different local densities are likely to correspond mainly to a different extent of polymerization. However, the comparison of the densities beside the TJ-network is limited by the fact that not all parts of the plasma membrane were similarly in focus during the SPDM measurements.

## Discussion

In this report, we demonstrate for the first time the analysis of TJ by localization microscopy. SPDM improves the optical resolution to values far below the diffraction limit of light microscopy using conventional fluorophores and standard fluorescent proteins such as YFP. This enables a detailed visualization of the network of TJ-strands, a quantitative analysis of the morphology of TJ, and provides a substantial improvement compared to conventional fluorescence microscopy. This method takes advantage of reconstituted TJ-strands which are oriented in xy-direction in the object plane. In contrast, TJ-strands in polar epithelial cells are oriented in the z-direction which impedes the application of the technique.

A recent publication reported the usage of EGFP for localization microscopy in a very similar way [19] to investigate the distribution of histones. In contrast to their approach of using a special “switching buffer” for achieving multiple transitions between a long-lived “Off” state and the “On” state of the fluorophores, we embedded the samples in a conventional embedding medium (Immu-Mount). As a result, the molecules remain in the “Off” state much longer (similar to a very early report about GFP/YFP “blinking” [20]) and most of them (∼88%) are detected only once during the measurement (Fig. S1). This provides the advantage of an improved analysis of the protein density and arrangement since no signal clusters are present in the image due to multiple detection of one single molecule. Nevertheless, all values for protein densities express only relative numbers since not every fluorophore is detected during the acquisition time.

Compared to the established freeze-fracture EM, localization microscopy (SPDM) has the following advantages: (i) direct detection of the YFP-labeled TJ-proteins, (ii) quantitative analyses of large TJ-networks, (iii) single molecule information for further statistical analyses.

The analysis revealed highly branched networks for Cld5- as well as for Cld3-strands reconstituted in HEK293 cells. This result is consistent with FF-EM analysis [5], [11], [21]. However, Cld3-strands reconstituted in L-fibroblasts showed less branching indicating additional cell type specific factors [22]. In contrast to these earlier studies, we provided a quantitative analysis of the mesh-formation of the strands. This revealed two types of meshes with a mean diameter of 100 nm or smaller and a mean diameter of 300 to 600 nm, respectively. Both types were found for Cld3 and Cld5. The two groups were separated more clearly for Cld5. The reason for the formation of these two groups is unclear. However, the two groups could be the result of a specific but yet undefined polymerization process which might be addressed in future studies.

Meshes with different diameters were also detected by FF-EM for Cld5- [5], Cld3- [11] and for Cld5/Cld3-cotransfected HEK293 cells (Fig. 3). Initial analysis of meshes formed by Cld5/Cld3 copolymeric TJ-strands and detected by FF-EM indicated mean diameters ([longest+shortest]/2) ranging from 80 to 650 nm (314±146 nm, mean±STD, n = 63). This suggests that copolymerization of Cld3 and Cld5 does not fundamentally change the diameter of the meshes. However, this should be analyzed in more details in subsequent morphometric studies using localization microscopy and FF-EM.

The Feret diameter for both claudins resulted in very similar distributions with just one peak at a value of ∼0.7. This suggests that a slightly elongated mesh is the sterically/energetically most stable condition of the mesh and that the extent of elongation can vary strongly. An analysis of the orientation of the elliptically shaped meshes yielded a correlation between the orientation of the single meshes and the whole TJ-network. The orientation of the meshes indicates that the shape of the meshes is determined by mechanical forces at the cell-cell contact rather than by intrinsic properties of the claudins. In the cells analyzed, the claudins were not specifically linked to the cytoskeleton [5]. However, in polarized cells containing the full TJ machinery, the flexibility in the orientation of the strands could be limited by zonula occludens proteins and other TJ-associated proteins [23]. In contrast, our data indicate that the strong branching and mesh formation of the strands is independent of association of the strands with the cytoskeleton.

With SPDM, not only polymeric claudin-strands but also non-polymerized claudins could be investigated with a structural resolution of 50 nm in a quantitative manner. This is a mayor advantage compared to other techniques like conventional fluorescence microscopy and FF-EM. It enables localization of distinct populations of claudin molecules; in particular, molecules in strands, molecules trapped in meshes, molecules outside of meshes close to strand network and molecules far away from networks. Estimation of the ratio of polymerized to non-polymerized claudins in a defined area of the plasma membrane provides information about the dynamics of TJ-assembly/disassembly. This could be combined with data obtained by FRAP to characterize the dynamics of TJ in more detail [9], [10], [11], [24].

The ultrastructure of Cld3- and Cld5-strands detected by FF-EM differs. Cld3 forms continuous strands on the protoplasmic face (P-face) of the plasma membrane, whereas Cld5 forms discontinuous strands (with 10–20 nm gaps) on the exoplasmic face (E-face) of the plasma membrane [4], [5], [11]. The molecular reason for this difference is unclear. The structural resolution achieved in the present SPDM measurements did not provide the means to resolve these fine structural details (Fig. S2). However, the gaps detected in FF-EM are likely to be due to the fact that claudins are imaged from one leaflet of the plasma membrane of one cell. In contrast, claudin-YFP molecules from both neighboring cells are detected with SPDM. This results in a similar appearance of Cld3- and Cld5-strands in this study. In addition, the density of detected molecules in the strands is also similar for both claudins. This result is important to note because it is consistent with the idea that Cld5 as well as Cld3 form continuous polymeric strands in the paracellular space and that the difference in continuity detected by FF-EM is a result of different molecular organization of Cld3- and Cld5-polymers. Interruptions or breaks (>50 nm) in the continuity of the strands were detected for Cld3 and Cld5 (Fig. 2, Fig. 7). These breaks are assumed to allow paracellular permeation of molecules [7], [8].

In sum, we can conclude that localization microscopy allows the quantification of the number of detected molecules, comparison of relative molecule densities, separation of polymeric and non-polymeric claudins, and quantification of morphological parameters of the TJ-strands. This technique can be expanded to the analysis of different TJ-proteins. For example, Cld1 and Cld11 are assumed to form much less meshes than Cld5. This could be efficiently quantified using SPDM and the introduced algorithm for the analysis of the mesh-like structures. Furthermore, CFP/GFP/YFP-tagged claudins can be coexpressed with non-tagged claudins or other TJ-proteins to analyze their effect on (i) the morphology of the strands formed by the tagged claudins, (ii) the relative density of claudins in the strands or (iii) the ratio of polymerized to non-polymerized molecules. Dual color imaging by SPDM is also possible [25], [26]. Therefore, coexpression of GFP/YFP or YFP/RFP pairs of TJ fusion proteins could enable colocalization studies with super-resolution. Taken together, localization microscopy based analysis as described in this study provides a unique tool to advance the molecular understanding of paracellular barriers.

## Materials and Methods

### Cell culture, labeling and specimen preparation

HEK293 cells were maintained in Dulbecco's modified Eagle's medium (DMEM, Invitrogen, Carlsbad, CA, USA) supplemented with 10% fetal calf serum, 100 units/ml penicillin and 100 µg/ml streptomycin (Invitrogen, Carlsbad, CA, USA). Transfections were performed as described previously [5]. Expression constructs for murine Cld3, Cld3-YFP and Cld5-YFP were described previously [5], [11]. HEK293 cell lines stably transfected with Cld3-EYFP or Cld5-EYFP were described previously [5], [11] and were maintained in the medium mentioned above supplemented with 0.25 mg/ml G418 (Calbiochem, La Jolla, CA, USA). Cells were plated on poly-L-lysine coated coverslips and three days later washed with PBS and then fixed by incubation with 2.4% paraformaldehyde, 100 mM Na-Cacodylat, 100 mM Sucrose, pH 7.5 for 10 min. Quenching was performed with 0.1 M glycine in PBS for 20 min. Cells were washed with PBS, incubated with 1% Bovine serum albumin in PBS for 10 min and mounted with Immu-Mount (Thermo Scientific, Pittsburg, PA, USA) on slides for microscopy.

### Setup for localization microscopy (SPDM)

Localization microscopy measurements were performed with a wide-field fluorescence microscopy setup according to Lemmer et al., 2008 [17]. This method allows optical isolation of single molecule signals utilizing a light induced long-lived dark state of the fluorophores [20], [27], [28], [29], [30]. Illumination of the sample with an excitation intensity in the 10 kW/cm^2^ range pushes the fluorophores within a few seconds into a long-lived dark state. The stochastic recovery of the molecules to the fluorescent state is then used for their optical isolation. Thus, localization microscopy can be performed with standard fluorophores, including standard fluorescent proteins like YFP.

For the present experiments a DPSS laser with a wavelength of 488 nm (Sapphire HP 488, Coherent, Dieburg, Germany) was used for image acquisition. No additional laser was needed for the switching/bleaching of the molecules. Fluorescent light was detected through a 1.4NA oil-immersion objective (HCX PL APO, 63×, Leica, Wetzlar, Germany) with a high quantum efficiency CCD camera (SensiCam QE, PCO Imaging, Kehlheim, Germany). A bandpass filter (XF3003 520DF40, Laser Components GmbH, Olching, Germany) was used to select the emitted fluorescence.

### Data acquisition and position determination

Image sequences consisting of 8,000 frames were recorded with an integration time of 55 ms. The positions of the single molecule signals were determined by the calculation of their center of mass without losing precision compared to iterative methods [31]. For a 2D Gaussian distribution without additional background as a model function the problem of maximizing the likelihood can be solved analytically. In the first step of the position determination signals are found by comparing each pixel value with the background 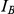 that is calculated by:
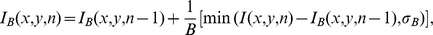
where 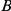 defines the inverse smoothing factor, 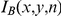 the pixel value at the position 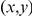 in the frame 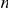 and 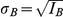 the width of the Poisson distributed background noise. After subtraction of the background all pixels are additionally reduced by 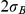 for the calculation of the molecules position 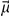. This suppresses very noisy pixels at the corners of the ROI around the single molecule signals. In the last step the position 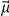 and the localization accuracy 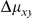 are determined:
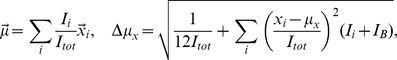
with the pixel value 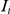 at the 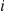-th pixel in the ROI and the total intensity of the signal 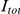. For more details see [31].

### Visualization of localization microscopy data

The mean localization accuracy of the measurements was ∼21 nm. The median of the distances to the next neighboring molecule positions was ∼10 nm. Due to the high density of detected molecules, the structural resolution in the localization microscopy image is mainly determined by the localization accuracy. For visualization of the data each molecule position was blurred by a Gaussian with a standard deviation corresponding to the mean value of the distance to the next four neighboring molecule positions. Also, the number of detected photons for each single molecule signal was considered for the visualization. Thus, an image was generated in which the intensity expresses the density of fluorophores as well as the amount of photons emitted by each individual molecule. This corresponds to the image formation in conventional fluorescence microscopy.

### Freeze-fracture electron microscopy

HEK cells were cotransfected with Cld3 and Cld5-YFP constructs, three days later, washed with PBS, fixed with 2.5% glutaraldehyde (electron microscopy grade, Sigma-Aldrich) in PBS for 2 h, washed and processed for freeze-fracture electron microscopy as reported [32].

## Supporting Information

Figure S1 **Blinking characteristics of YFP in standard embedding medium.** Histograms of the number of neighboring molecules within a radius of 20 nm around each detected molecule. The yellow distribution shows the result of a SPDM measurement of a homogenously YFP-labeled plasma membrane of a SKBr3 cell. The red distribution results from a simulation with the same amount of points and the same mean density of points as in the SPDM measurement assuming that every molecule was detected only once. Simulated data, assuming that 88% of all molecules are detected once and 12% twice or more (shown in green), agrees very well with the experimental data.
(TIF)

Figure S2 **Simulations for localization microscopy images of TJ-strands.** The simulations are based on lines with a width of 10 nm. Gaps of different sizes (ranging from 10–100 nm) were included. For the generation of the localization microscopy images all parameters were set according to those of the actual SPDM measurements. Therefore, a localization accuracy of 20 nm was assumed. The amount of points in the image was set a value resulting in a mean distance to the next neighboring point of 10 nm, including a background of randomly distributed points with a mean density of 400 points/µm^2^. In **A** the positions of the individual points scattering around the white lines are represented by red crosses. **B**: simulated localization microscopy image visualized in the same way as the images of the experimental data. Due to the localization accuracy the structural resolution is limited to ∼50 nm. This can also be observed in **B**. Here the gaps ≥50 nm can be resolved. Statistical fluctuations in the detection of the molecules result in fluctuations of the point density along the strands.
(TIF)

Figure S3 **Analysis of the orientation of the meshes with respect to the orientation of the whole TJ-network.** The dimensions of the meshes (individual meshes given in different colors) in the direction of the TJ-network were determined as well as the dimension in the perpendicular direction. Only meshes with a mean diameter >200 nm were included in the analysis. The ratio between the dimensions of the meshes in the direction of the TJ-networks and the dimensions in the perpendicular direction was for both claudin types around 1.25.
(TIF)
