# Supplementary figures and images for: Visualization and Quantitative Analysis of Reconstituted Tight Junctions Using Localization Microscopy
Source: PLoS One. 2012 Feb 2;7(2):e31128. doi: 10.1371/journal.pone.0031128 (PMC3271094; doi:10.1371/journal.pone.0031128)

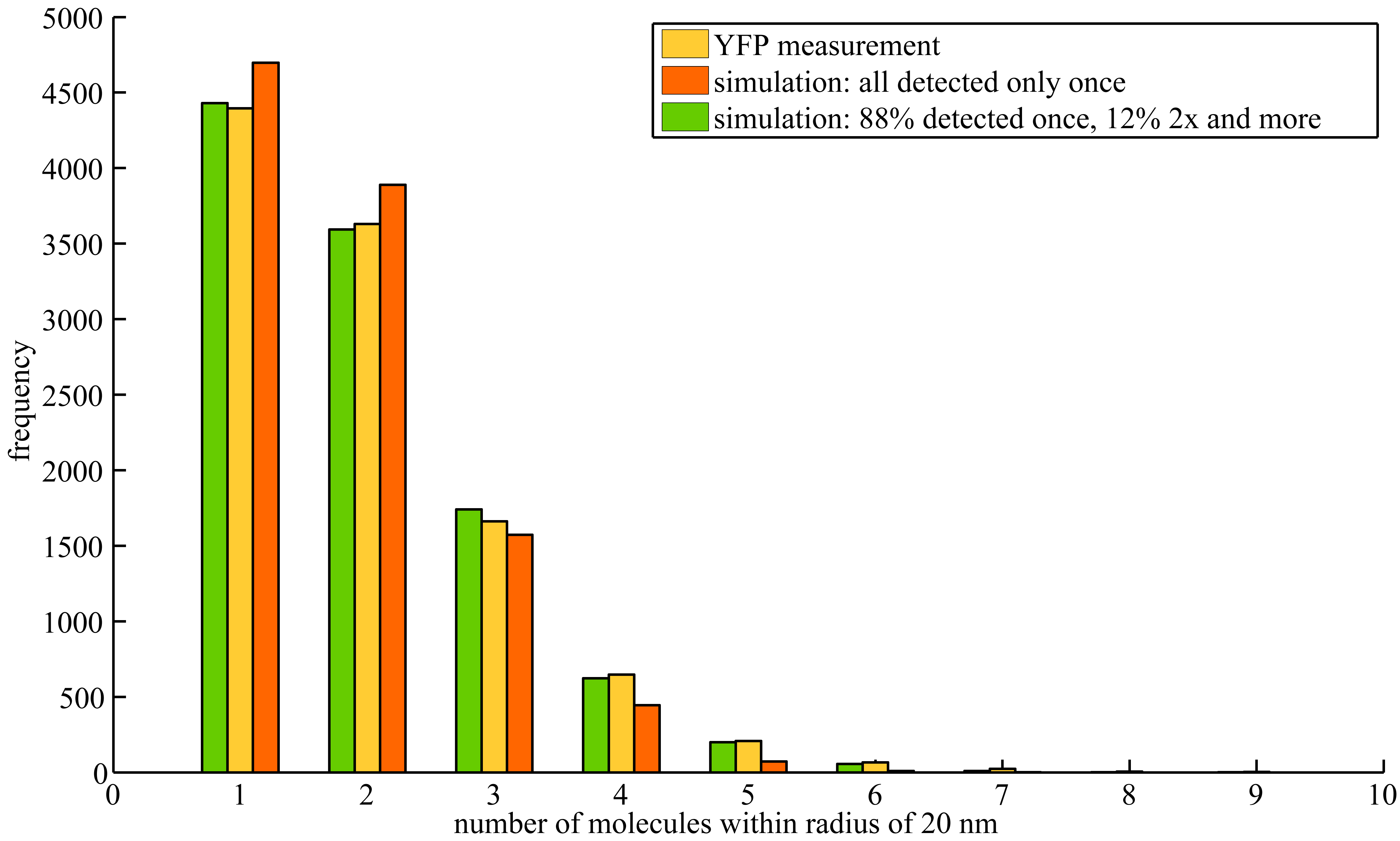

Supplement: Figure S1 — Blinking characteristics of YFP in standard embedding medium. Histograms of the number of neighboring molecules within a radius of 20 nm around each detected molecule. The yellow distribution shows the result of a SPDM measurement of a homogenously YFP-labeled plasma membrane of a SKBr3 cell. The red distribution results from a simulation with the same amount of points and the same mean density of points as in the SPDM measurement assuming that every molecule was detected only once. Simulated data, assuming that 88% of all molecules are detected once and 12% twice or more (shown in green), agrees very well with the experimental data. (TIF) [file pone.0031128.s001.tif]

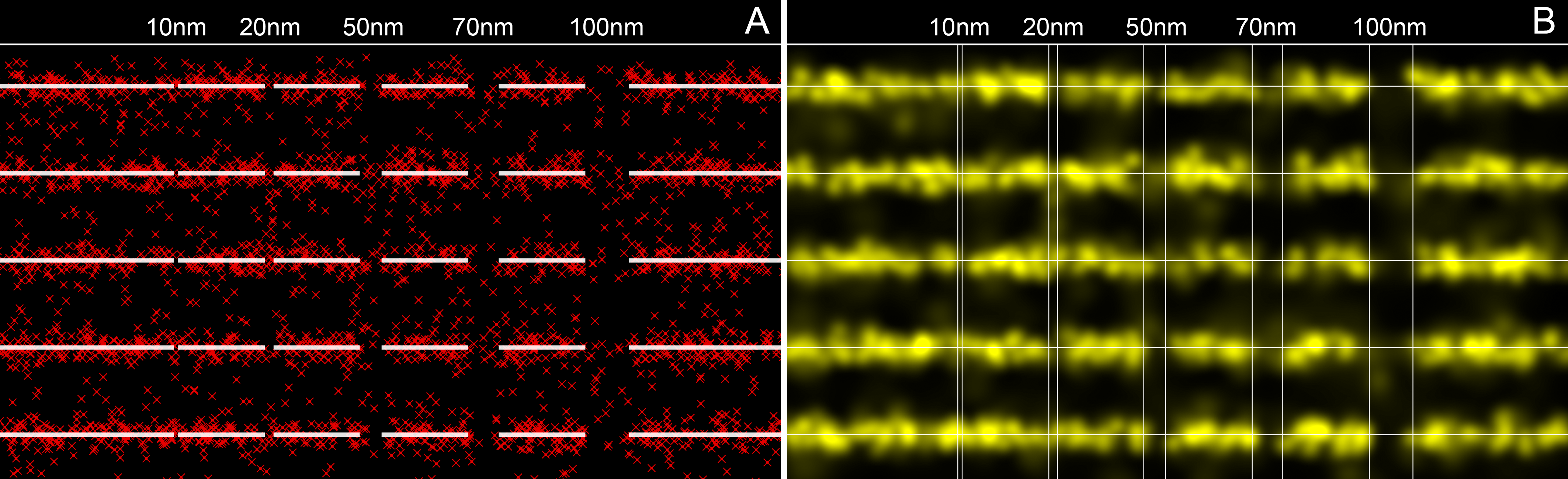

Supplement: Figure S2 — Simulations for localization microscopy images of TJ-strands. The simulations are based on lines with a width of 10 nm. Gaps of different sizes (ranging from 10–100 nm) were included. For the generation of the localization microscopy images all parameters were set according to those of the actual SPDM measurements. Therefore, a localization accuracy of 20 nm was assumed. The amount of points in the image was set a value resulting in a mean distance to the next neighboring point of 10 nm, including a background of randomly distributed points with a mean density of 400 points/µm2. In A the positions of the individual points scattering around the white lines are represented by red crosses. B: simulated localization microscopy image visualized in the same way as the images of the experimental data. Due to the localization accuracy the structural resolution is limited to ∼50 nm. This can also be observed in B. Here the gaps ≥50 nm can be resolved. Statistical fluctuations in the detection of the molecules result in fluctuations of the point density along the strands. (TIF) [file pone.0031128.s002.tif]

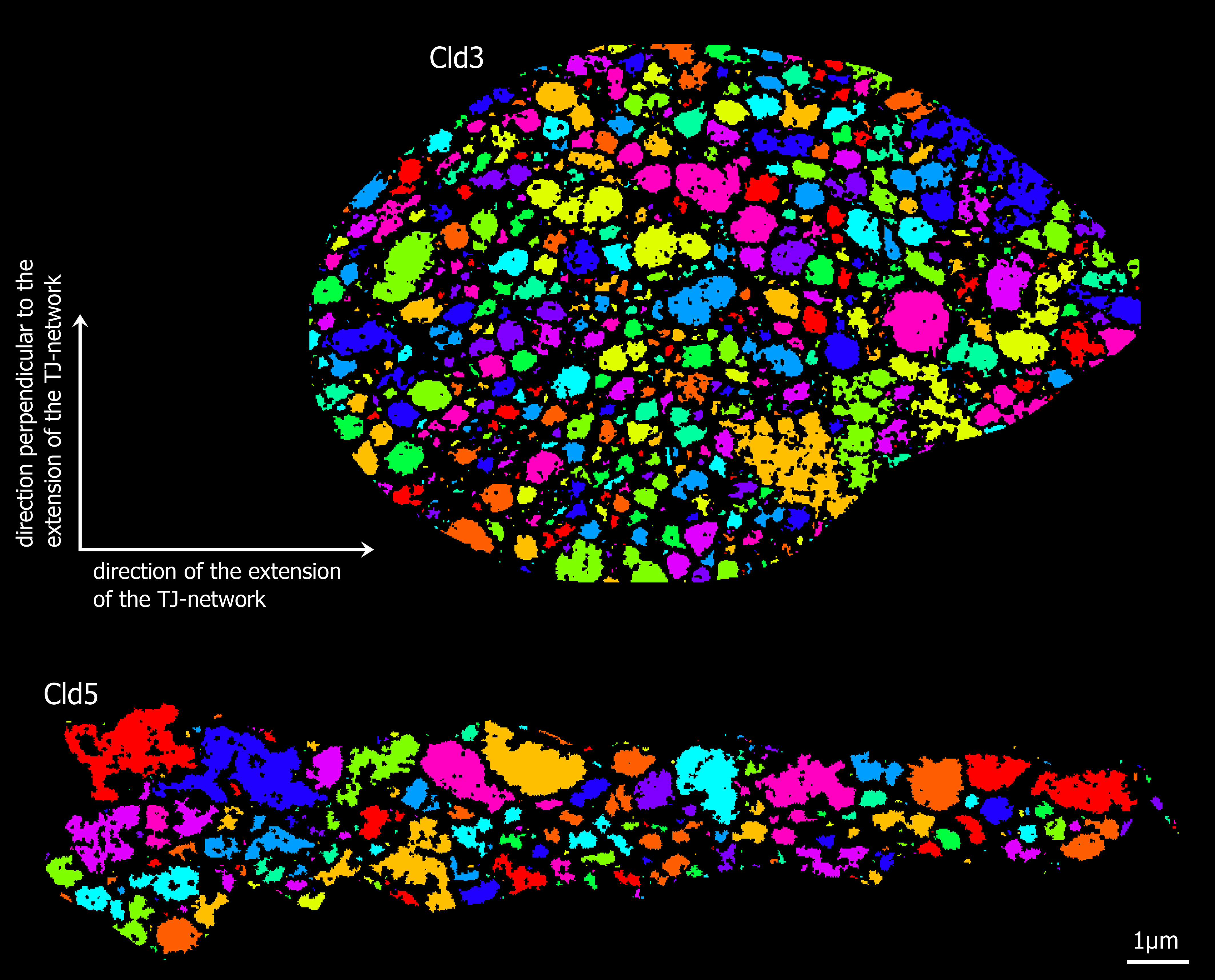

Supplement: Figure S3 — Analysis of the orientation of the meshes with respect to the orientation of the whole TJ-network. The dimensions of the meshes (individual meshes given in different colors) in the direction of the TJ-network were determined as well as the dimension in the perpendicular direction. Only meshes with a mean diameter >200 nm were included in the analysis. The ratio between the dimensions of the meshes in the direction of the TJ-networks and the dimensions in the perpendicular direction was for both claudin types around 1.25. (TIF) [file pone.0031128.s003.tif]
